# Pregnancy Considerations in the Multidisciplinary Care of Patients with Pulmonary Arterial Hypertension

**DOI:** 10.3390/jcdd9080260

**Published:** 2022-08-11

**Authors:** Julie Coursen, Catherine E. Simpson, Monica Mukherjee, Arthur J. Vaught, Shelby Kutty, Tala K. Al-Talib, Malissa J. Wood, Nandita S. Scott, Stephen C. Mathai, Garima Sharma

**Affiliations:** 1 Department of Medicine, Johns Hopkins University, Baltimore, MD 21218, USA; 2 Divisions of Pulmonary and Critical Care Medicine, Department of Medicine, Johns Hopkins University, Baltimore, MD 21218, USA; 3 Division of Cardiology, Department of Medicine, Johns Hopkins University, Baltimore, MD 21218, USA; 4 Division of Maternal Fetal Medicine, Department of Gynecology Obstetrics, Johns Hopkins University, Baltimore, MD 21218, USA; 5 Division of Cardiology, Department of Medicine, Massachusetts General Hospital, Boston, MA 02114, USA

**Keywords:** pulmonary hypertension, pregnancy, peripartum cardiovascular disease, cardio-obstetrics

## Abstract

Pulmonary arterial hypertension (PAH) is a vasoconstrictive disease of the distal pulmonary vasculature resulting in adverse right heart remodeling. Pregnancy in PAH patients is associated with high maternal morbidity and mortality as well as neonatal and fetal complications. Pregnancy-associated changes in the cardiovascular, pulmonary, hormonal, and thrombotic systems challenge the complex PAH physiology. Due to the high risks, patients with PAH are currently counseled against pregnancy based on international consensus guidelines, but there are promising signs of improving outcomes, particularly for patients with mild disease. For patients who become pregnant, multidisciplinary care at a PAH specialist center is needed for peripartum monitoring, medication management, delivery, postpartum care, and complication management. Patients with PAH also require disease-specific counseling on contraception and breastfeeding. In this review, we detail the considerations for reproductive planning, pregnancy, and delivery for the multidisciplinary care of a patient with PAH.

## 1. Introduction

Pulmonary arterial hypertension (PAH) is a chronic pulmonary vascular syndrome caused by pathologic vasoconstriction and endothelial dysfunction of the distal pulmonary arteries (PA) leading to increased afterload and, if untreated, progressive right heart remodeling and failure [1,2,3]. Given the complex hemodynamic derangements seen in PAH, further hemodynamic changes that occur with pregnancy in patients with PAH are associated with high morbidity and mortality. Due to these risks, international consensus guidelines from the European Society of Cardiology (ESC) and European Respiratory Society (ERS) [4], Pulmonary Vascular Research Institute [5], and American College of Cardiology Foundation/American Heart Association [6] recommend against pregnancy in patients with PAH. Most modern studies estimate maternal mortality for pregnant PAH patients at 9–17% [7,8,9,10,11,12,13,14]. Patients with severe PAH have worse hemodynamic changes and outcomes compared to those with milder disease [7,9,11,14,15,16], and estimates of maternal mortality in severe PAH can be 36% or higher [17,18]. However, a number of single institution reports with limited sample sizes have demonstrated improved maternal outcomes [19,20,21,22,23,24,25,26], particularly in patients with milder disease [27], highlighting the value of risk stratification and shared decision making in reproductive planning as well as the evolving nature of this area of study. Several neonatal complications are also well reported in the literature, including small for gestational age infants, preterm birth, and fetal mortality [28]. In this review, we describe the physiological changes during pregnancies of patients with PAH, explain the classification of pregnancy in PAH based on risk prediction tools, and focus on reproductive planning, pregnancy, and delivery considerations in the multidisciplinary care of patients with PAH.

## 2. Comment on Dobbs V. Jackson Women’s Health Organization

International pulmonary consensus guidelines, as detailed in this review, recommend termination for pregnant patients with PAH due to the high risk for morbidity and mortality [5]. After the United States (U.S.) Supreme Court released their decision in the Dobbs v. Jackson Women’s Health Organization [29] case, removing a Constitutional right to abortion and reversing Roe v. Wade and Casey v. Planned Parenthood decisions, the American College of Obstetricians and Gynecologists (ACOG), American Academy of Family Physicians (AAFP), American Academy of Pediatrics (AAP), American College of Physicians (ACP), and American Psychiatric Association (APA) condemned the decision and affirmed the importance of safe, legal abortive options [30,31]. As the federal right to abortion has been overturned in the U.S., this requires more engagement from providers to ensure that women with elevated risk have adequate contraception. This review addresses the medical considerations of pregnancy, including termination, during PAH and does not address any associated legal issues.

## 3. Background

**Figure 1:**
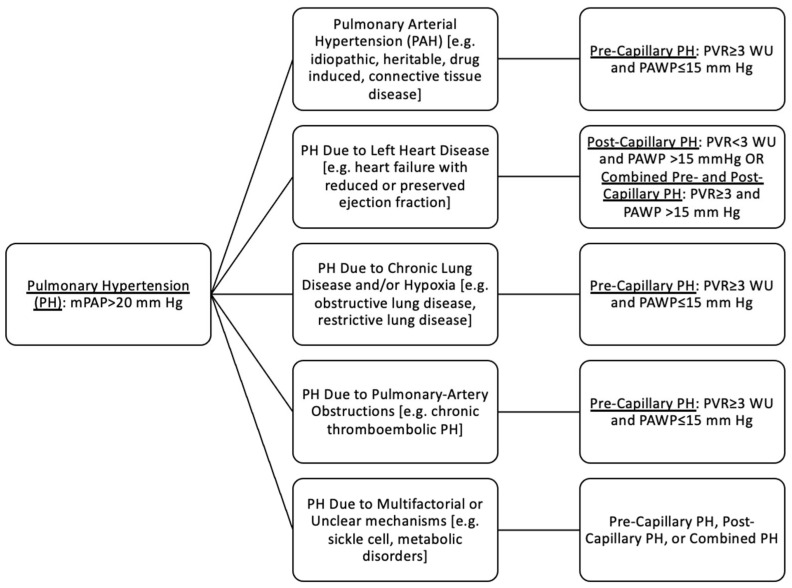
Classification of pulmonary hypertension. mPAP = mean pulmonary artery pressure. PVR = pulmonary vascular resistance. PAWP = pulmonary arterial wedge pressure. WU = Wood units.

Definition: As defined by the World Symposium on Pulmonary Hypertension (WSPH) in 2018, pulmonary hypertension (PH) is a mean pulmonary artery pressure (mPAP) greater than 20 mm Hg measured during resting right heart catheterization [32]. PH can be classified into five clinical groups, grouped by associated etiologies, treatment responses, histopathologic findings, and hemodynamic characteristics [2] (Figure 1). PAH is pre-capillary pulmonary hypertension, characterized by mPAP greater than 20 mm Hg, pulmonary vascular resistance (PVR) greater than 3 Wood units, and pulmonary arterial wedge pressure (PAWP) less than 15 mm Hg, in the absence of significant left heart disease, lung disease, or thromboembolic disease [32].

Risk Assessment: Cardiovascular risk assessment tools for pregnant patients, including the Modified World Health Organization (mWHO) classification [33] and Cardiac Disease in Pregnancy CARPREG II Risk [34], categorize patients with PH as high-risk for cardiovascular complications (Table 1). The mWHO classification for PH is category IV [33], which indicates the risk of maternal cardiovascular complications is 40–100% [35]. The CARPREG II risk score estimates a 10% risk of maternal cardiac complications for patients with PH [34].

Risk assessment and stratification is recognized as a valuable predictor of survival outcomes for patients with PAH [36], and the ESC/ERS guidelines recommend a goal-directed treatment approach to induce milder or lower risk disease [4,37]. As outlined by the ESC/ERS guidelines, high-risk PAH is defined by several clinical, laboratory, imaging and hemodynamic findings, including right heart failure, rapid progression of symptoms, recurrent syncope, a 6-min walk distance of less than 165 m, NT-proBNP greater than 1400 ng/L, and other findings on echocardiogram, cardiopulmonary exercise testing, and right heart catheterization [4]. The World Health Organization Functional Class (WHO FC), which was adapted from New York Heart Association heart failure classes [38], is also part of this assessment [4]. Patients with WHO FC IV have symptoms of right heart failure, such as dyspnea or fatigue at rest and worsened with exertion, and are associated with worse prognosis compared to other functional classes [39]. Based on comprehensive assessment with these clinical, functional, and hemodynamic measurements, risk stratification to discriminate level of severity is accurate in predicting prognosis for patients with PAH [40,41,42]. Tools for risk stratification are available from the European Cardiology Society/European Respiratory Society Guidelines [36], Swedish Pulmonary Arterial Hypertension Register [40], the Comparative, Prospective Registry of Newly Initiated Therapies for Pulmonary Hypertension [43], the French Pulmonary Hypertension Registry [41], and the Registry to Evaluate Early and Long-Term PAH Disease Management (REVEAL, as well as the revised REVEAL 2.0) [44,45]. Because of its usefulness in predicting survival for patients with PAH [46,47] and refined discrimination in risk stratification compared to other tools [45], the REVEAL risk calculator is preferred at our institution. Risk assessment is also continuously assessed throughout pregnancy, as it is dynamic with clinical disease.

Given there are worse maternal and fetal outcomes reported with severe disease [7,9,11,14,15,16], these risk assessments are valuable in estimating outcomes and prognosis, including during pregnancy. Therapeutic abortion and premature delivery have a higher incidence in patients with severe PAH compared to milder disease [7]. Offspring outcomes, such as small-for-gestational age and fetal death, are higher for patients with severe PAH compared to mild PAH [7]. Risk stratification is valuable when counseling patients during reproductive planning.

### 3.1. Physiological Changes in Pregnancy and Pulmonary Arterial Hypertension

**Figure 2:**
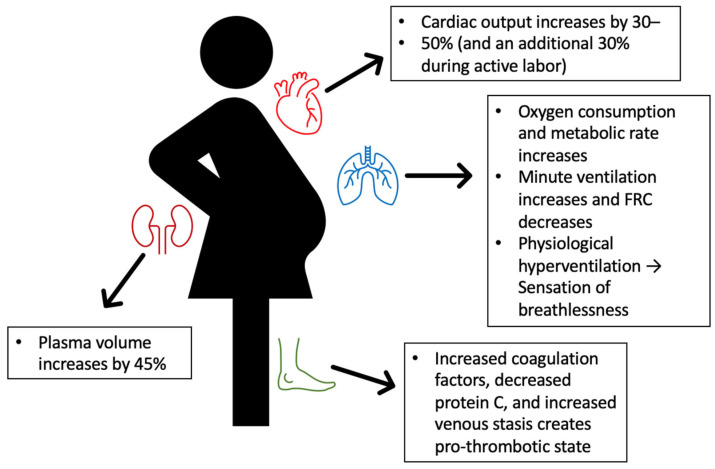
Physiological changes during pregnancy.

There are significant physiological changes in every organ system during pregnancy to accommodate the growing fetus (Figure 2). These physiological changes during pregnancy can exacerbate underlying cardiovascular disease [35] or unmask previously occult PAH [48,49]. Pregnancy has even triggered PAH in a previously healthy patients with genetic predisposition (*BMPR2* gene mutation) [50].

Patients with PAH are particularly vulnerable to hemodynamic changes and fluid shifts. Hemodynamic changes in pregnancy are driven primarily by cardiac output, which increases ~45% above pre-pregnancy baselines [51]. In the respiratory system, pregnancy causes decreased functional residual capacity and increased minute ventilation, resulting in physiological hyperventilation, which can create a sensation of breathlessness [52]. PVR decreases during pregnancy, and PAWP and central venous pressure remain stable, despite the rise in volume [35]. Plasma volumes increase ~45% more than pre-pregnancy states [53], and these volumes peak predelivery [35]. Increases in plasma volume can stretch cardiac chambers, resulting in worsened valvular regurgitation, and increase afterload on the heart [35]. Pregnant patients also experience sympathetic activation with increases in heart rate up to 25% above baseline [51], increases in metabolic rate by ~15%, and increases in oxygen consumption by ~20% [54]. Systemic vascular resistance decreases during pregnancy, then increases with delivery and postpartum [53]. For patients with PAH, increases in RV contractility may be insufficient to meet progressive increases in afterload, especially immediately postpartum, resulting in RV-PA uncoupling and overt right heart failure [1].

Hormonal changes can also strain on the cardiopulmonary system. Hormone-mediated vasodilation from progesterone leads to activation of the renin-angiotensin-aldosterone system (RAAS) [51]. RAAS activation leads to further retention of sodium and volume, which has the potential to overwhelm a vulnerable RV. Estrogen increases during pregnancy [54], but sex hormones like estrogen have a complex, poorly understood relationship in PAH, which has been deemed the “estrogen puzzle” or paradox [55]. Estrogen likely causes compartment-specific effects, as suggested by their positive effect on the RV [56,57] but deleterious effects on pulmonary circulation remodeling [58]. The overall clinical impact of estrogen in PAH during pregnancy is unclear.

Pregnancy leads to coagulation changes, including the development of a pro-thrombotic state due to increased circulating coagulation factors and acquired protein C resistance [59]. Inferior vena caval compression by the gravid uterus leads to lower extremity venous stasis, further increasing the risk for thromboembolism [35]. In this pro-thrombotic state, a provoked pulmonary embolism could trigger an acute, lethal increase in PA pressures with diseased right heart and pulmonary vasculature.

In sum, the physiological changes required for pregnancy are taxing on the diseased pulmonary vasculature and right heart. The most critical times in the pregnancy are the times of greatest hemodynamic changes, particularly mid trimester fluid shifts (especially weeks 20–24) [17], during active labor and delivery secondary to Valsalva, and the immediate post-partum periods when there is auto-circulation of about 300–500 cc of uterine blood back into the maternal circulation [60].

### 3.2. Reproductive Planning and Contraceptive Counseling Considerations in Patients with PAH

Preconception counseling: Due to the high risk of maternal and fetal/neonatal complications, patients with PAH should be counseled on the risks of pregnancy. Patients with severe PAH are generally counseled against pregnancy, and for those who become pregnant, termination is considered, as recommended by consensus pulmonary guidelines from the Pulmonary Vascular Research Institute [5]. This discussion is especially relevant for patients with severe pulmonary hypertension or signs of RV failure, as mortality is linked to RV performance and hemodynamics prior to pregnancy [16]. For patients with PAH who decide to continue pregnancy, the hemodynamic changes of pregnancy can worsen once quiescent cardiovascular symptoms. Pregnancy has the potential to transition a well-controlled PAH patient to more severe disease, which is associated with higher mortality and in which case, second trimester therapeutic termination can be considered [17]. For those that wish to become pregnant despite the risks, they are counseled on teratogenic medications, as follows, and the benefits of milder, well-controlled disease prior to pregnancy.

Contraception counseling and reproductive planning is standard of routine care for patients with PAH [61,62,63]. This is especially pertinent, as PAH is more prevalent in women of childbearing age [47,64,65,66]. An assessment of each individual’s contraception risks (e.g., thromboembolism risk, bleeding profile, adherence, and side effect profile), barriers to contraceptive access, personal preferences (e.g., reversibility), and reproductive goals should be completed to inform patient counseling [62].

Preconception counseling can be provided prior to medication initiation to avoid inadvertent teratogenic exposure before recognition of early pregnancies [67]. Preconception counseling can also include discussion of fertility preservation options, such as oocyte or sperm cryopreservation, prior to initiation of teratogenic medications; however, this practice may be limited due to the risks of general anesthesia during oocyte retrieval (risks of anesthesia further discussed in Anesthesia section).

Permanent irreversible contraception counseling: For patients with severe, non-reversible disease, permanent contraception is often discussed and recommended. Hysteroscopic sterilization is the preferred approach to minimize procedural risks. Mini-laparotomy can be considered for tubal ligation [5], but there are perioperative risks associated with general anesthesia (risks of anesthesia further described in Delivery section). A laparoscopic approach for tubal ligation is generally less favored due to potential procedural risks [5].

Hormonal contraceptive counseling: Estrogen-containing options (combined estrogen-progestin oral contraceptive, transvaginal ring, contraceptive patch) and injectable progestin increase the risk of venous thromboembolism [68], which could be fatal with a vulnerable or dysfunctional RV. Estrogen-containing contraceptives are considered a WHO Class IV for risk classification of contraceptives, indicating the method represents an unacceptable health risk for PAH and pulmonary vascular disease [61]. Therefore, these options are generally discouraged and avoided in patients with PAH. However, low-dose estrogen-containing options have been considered when concomitant with anti-coagulation [69]. Progestin-only oral options do not carry the same pro-thrombotic risk, but they require stricter adherence for efficacy in pregnancy prevention and are therefore not generally recommended [70]. Barrier methods are safe, but generally not recommended due to higher risk of failure (~18–28% 1-year failure rate with typical use) [62]. To ameliorate the reduced efficacy as monotherapy, barrier methods are sometimes recommended in combination with progesterone options [71]. The endothelin receptor antagonist bosentan reduces the efficacy of oral contraceptive pills and progestogen implant, so these methods should not be used as contraceptive monotherapy [68,70].

Long-acting reversible contraceptive (LARC): Copper and progestin-only intrauterine devices (IUDs) are generally the most preferred method for women with cardiovascular disease due to their longevity, effectiveness, and limited procedure risk of adverse events [62]. IUDs carry a theoretical risk of vasovagal response with insertion, so care should be taken to minimize discomfort during placement. Inpatient monitoring during placement can be considered for patients with severe disease [70].

### 3.3. Pregnancy Counseling in Patients with PAH

Termination: Due to the high risk of maternal decompensation and fetal/neonatal complications, patients who become pregnant with severe PAH are often counseled to pursue termination [5]. These decisions can be carefully considered with shared decision making and individual risk stratification, especially given the progressing nature of the field and improving outcomes with milder disease [27]. For patients who choose to pursue termination, the abortion procedure is generally safe with fewer risks than pregnancy [72]. Once decided, it is beneficial to pursue termination without delay, as risk increases with pregnancy duration [73]. For emergency hormonal contraception, it is notable that bosentan reduces the efficacy due to drug-drug interaction [68,70]. Patients with severe disease and higher risks can be monitored inpatient at tertiary care centers with established Pregnancy Heart Teams during termination. Particular attention may be given to higher risk patients, including those with second trimester pregnancies and those on anticoagulation [62].

Early pregnancies can be medically aborted with the synthetic prostaglandin mifepristone or misoprostol [33,74]; however, heart rate and blood pressure are generally monitored closely due to risk of medication-induced decreases in systemic vascular resistance. Medical and surgical abortions have similar rates of complications, but surgical abortion may be favored for patients at high risk of needing operative evacuation [74]. Saline abortions are generally not favored due to risk of increasing intravascular volume [33].

Pregnancy: After counseling on the potential risks, some patients may elect to proceed with pregnancy. In fact, pregnancy in PAH is increasing over time [75]. For patients with known heritable or idiopathic PAH, genetic counseling can be offered prior to conception [5]. Patients with cardiovascular disease, including those with PAH, are recommended for early referral to a specialist center with a multidisciplinary, collaborative cardio-obstetrics team [76] and experience in managing PAH in pregnancy (further discussed in Delivery section). Delay in referral for pregnant patients with cardiovascular disease is a known cause of preventable maternal mortality [77]. Through the multidisciplinary care team, a pregnancy care plan can be outlined from early on, including timing and mode of delivery for the woman with PAH (further discussed in Peripartum Planning section).

Patients should receive regular outpatient counseling and monitoring with a pulmonary hypertension/cardio-obstetric team. Consensus pulmonary guidelines recommend monthly appointments during first and second trimesters and weekly appointments during third trimester [5]. At our institution, typical practice includes patient appointments every other week during the first and second trimester and every week during the third trimester. Appointments are with the primary PAH physician (pulmonology or cardiology) and maternal fetal medicine. Disease progression can be monitored through evaluation of WHO functional class, 6-min walk test (6MWT), cardiopulmonary exercise testing, BNP/plasma NT-proBNP, and echocardiograms [78]. At our institution, a 6MWT is completed at initial evaluation, and an echocardiogram is completed each trimester or with change in clinical symptoms. Both BNP and plasma NT-proBNP are useful in clinical practice, but NT-proBNP has the benefit of a longer half-life compared to BNP [79].

### 3.4. Medication Counseling during Pregnancy in Patients with PAH

Calcium-Channel Blockers: Calcium channel blockers are safe during pregnancy [80], but their effectiveness is limited to those with the responder phenotype as seen on acute vasoreactivity testing during right heart catheterization [4,81]. The Food and Drug Administration (FDA) labels amlodipine as a Category C medication, indicating no adequate, well-controlled studies have been completed during pregnancy [82]. Unfortunately, patients also often demonstrate a time-limited response to calcium channel blockers, and only ~6.8% of patients are chronic responders [69]. Thus, even though calcium channel blockers are safe for use in pregnancy, they should not be used as substitutes or alternative therapy for PAH patients who do not have an established responder phenotype. Calcium channel blockers should also be avoided in patients with WHO functional class IV or evidence of RV impairment. Patients on calcium channel blocker monotherapy should be monitored closely for deterioration, as combination therapy is considered superior to monotherapy for clinical outcomes [83].

Phosphodiesterase-5 Inhibitors: Phosphodiesterase-5 inhibitors are acceptable, safe medications during pregnancy [8,9,84]. The FDA labels sildenafil as a Category B medication [85], indicating it is likely safe to use during pregnancy. Phosphodiesterase-5 inhibitors are most appropriate for patients with WHO functional class I or II and normal RV function. If there are any concerns for poor absorption, such as post-operative ileus, patients can be transitioned from oral to IV formulation of phosphodiesterase-5 inhibitors [5]. Oral phosphodiesterase-5 inhibitors can also be safely combined with parenteral prostaglandin therapy during pregnancy [26,84,86].

Prostaglandins: Prostaglandins are safe during pregnancy [19,87,88,89]. Prostaglandins improve RV function and hemodynamics by reducing pulmonary artery pressure and inhibiting abnormal platelet aggregation to promote improved pulmonary endothelial cell function [90]. Patients with WHO functional class III can be treated with inhaled prostaglandin, such as iloprost [19]. For patients with WHO functional class IV or evidence of severe RV impairment, they can be treated with parenteral prostaglandin, such as IV epoprostenol [91]. Treprostinil, which is preferred at our institution due to its stability at room temperature and longer half-life compared to IV epoprostenol, is an FDA Category B medication during pregnancy [92]. Subcutaneous treprostinil is another option, but side effects include adverse site reactions, which has historically limited its use [93]. Selexipag, an oral prostacyclin receptor agonist, did not demonstrate teratogenicity in pre-clinical trials, but its manufacturer does not recommend use during pregnancy due to the unknown nature of its teratogenic potential in humans [94]. Escalation therapy can include a combination of phosphodiestase-5 inhibitor and parenteral prostaglandin [5].

Endothelial Receptor Antagonists and Soluble Guanylate Cyclase Stimulators: Endothelial receptor antagonists (bosentan, macitentan, ambrisentan) and soluble guanylate cyclase classes (riociguat) have a significant risk for fetal birth defects and are immediately discontinued during pregnancy [95,96]. Patients with PAH should be counseled on the importance of longitudinal, prospective family planning, so contraindicated medications can be transitioned to safe alternatives prior to pregnancy. The FDA recommends discontinuation of bosentan, macitentan, ambrisentan, and riociguat at least one month prior to attempts at conception [97,98,99,100]. Ambrisentan and riociguat are Category X medications during pregnancy [97,99].

Diuretics: Diuretics like furosemide and torsemide are acceptable, but spironolactone should be minimized due to its risks of anti-androgenic effects on the fetus, particularly during the first trimester [68]. However, if the fetus is female, these risks may be abated.

Anti-coagulation: Anti-coagulation may provide survival benefit for patients with idiopathic PAH, but its benefit is unclear in other forms [101]. Shared decision making and individual risk assessment is used to determine anti-coagulation in other forms of PAH [2]. Pregnant patients taking anticoagulation can be transitioned to unfractionated or low molecular weight heparins for safety during pregnancy. Warfarin is generally discontinued; however, it notable that low doses of warfarin (≤5 mg) are considered acceptable in some pregnant patients with valvular disease [102]. Direct oral anticoagulants are currently not well studied in PAH [101]. Prophylactic heparin can be administered in the peripartum period. For patients transitioning to heparin or low molecular weight heparin, Factor IXa levels ensure appropriate dosing for full anti-coagulation [103].

### 3.5. Delivery and Role of Multidisciplinary Care

Peripartum Planning: Labor and delivery is a time of greater risk for morbidity for patients with PAH [17], particularly those with severe disease, but outcomes are cautiously more optimistic for patients with well-controlled, mild disease [27]. Indicators of high-risk for decompensation include severe right heart dysfunction, severe PH (such as mPAP > 50), Eisenmenger syndrome, and acute heart failure [104]. Given the dynamic nature of clinical disease, risk stratification can be reassessed repeatedly during peripartum planning. Risk assessment can be used to inform delivery planning and execution, allowing for clinical judgment to escalate care and monitoring as needed with more severe disease.

**Figure 3:**
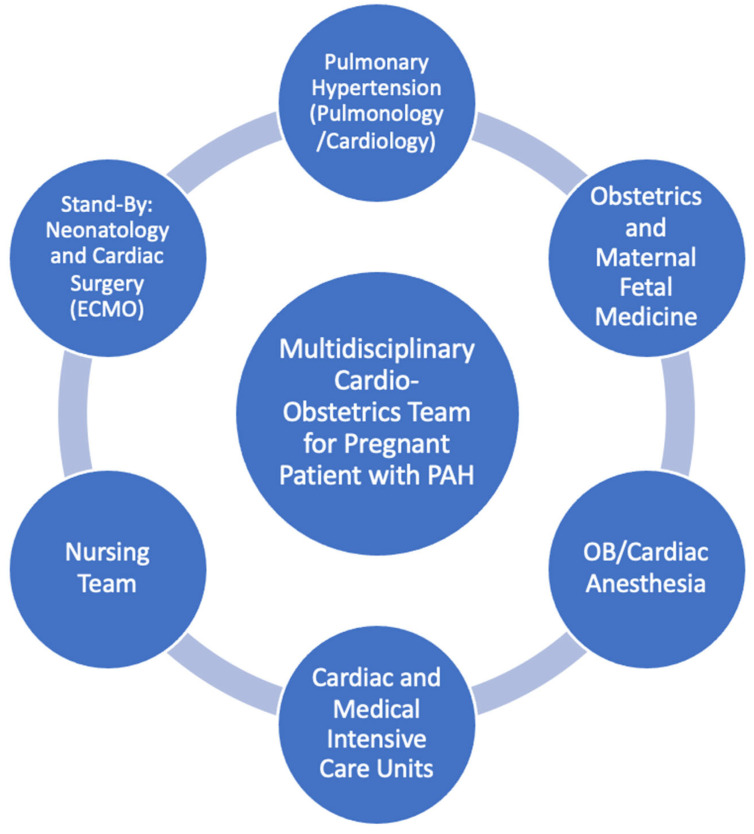
Multidisciplinary cardio-obstetrics care team for pregnant patient with PAH. ECMO = extracorporeal membrane oxygenation. OB = obstetrics.

Team coordination can be undertaken in advance through the pregnancy care plan. Delivery is generally completed in consultation with a multidisciplinary team, including PAH specialists in pulmonology, cardiology, maternal fetal medicine (MFM), and cardiac and/or obstetric anesthesiology [21,23,70]. The nursing team is also instrumental in physician/patient support, management, and resource/medication delivery [76]. Additional specialists may be needed on standby, such as cardiothoracic surgery in case of need for veno-arterial extracorporeal membrane oxygenation (ECMO) support, and neonatologists, in case of preterm delivery [21]. The specialist center should include a medical and/or cardiac intensive care unit (Figure 3).

At our institution, after a pregnant patient with PAH is identified, a multidisciplinary meeting is convened during the first trimester of pregnancy with subspecialists in pulmonology, cardiology, MFM/obstetrics, cardiac and obstetric anesthesia. These stakeholders convene routinely throughout the pregnancy, as follows. At the first meeting, the goal is to evaluate and develop consensus regarding the health of patient and fetus, severity of PAH, and identification of any other medical or social issues that are important in the care of the patient during the perinatal period. The next meeting is convened during the second trimester to review the course of the pregnancy and begin considering and planning for delivery scenarios. In the third trimester, as the preferred window for delivery as determined by gestational age or other patient factors (such as comorbidities or complications during pregnancy) approaches, a preferred delivery scenario is selected. Prior to the patient’s planned hospitalization, a simulation of the delivery workflow is completed to ensure all logistical considerations have been addressed. The multidisciplinary team then meets again within 48 h of the planned delivery, on the day of delivery, postpartum day 1, and on day of discharge to address clinical issues as they arise in real time.

Delivery: The decision of vaginal versus Cesarean delivery (C-section) requires careful clinical judgement because there are risks with each method (Table 2), but consensus pulmonary guidelines currently recommend C-section [5]. General principles of delivery include avoiding increases in PVR and RV afterload, maintaining stable systemic blood pressure and venous return without rapid fluid shifts, and minimizing myocardial depressants [21]. Timing of induction for planned delivery requires consideration about the stability of mother and fetus; however, 34–36 weeks is usually deemed acceptable for a planned and scheduled delivery [21,70].

Vaginal Delivery: Vaginal delivery has the potential risk of pain, which, if poorly controlled, can precipitate a vasovagal spiral with catecholamine release, hypoxia, and acidosis, resulting in worsening pulmonary vasculature tone and RV function [105]. Alternatively, pain control through overly aggressive opiate management can lead to hypercapnia and respiratory acidosis [106]. Additionally, to compensate for the increased stress of active labor, cardiac output increases by ~30% [35], which can overwhelm a vulnerable RV.

There are, however, reports of successful vaginal delivery in PAH. A single center study reported improved mortality through planned induction, peripartum IV prostacyclin, and modified vaginal delivery with limited Valsalva pushing using only abdominal muscles to avoid vasovagal response [21]. Modified pushing has also been known as the “cardiac vaginal delivery” due to its benefits in avoiding the rapid preload reduction seen in Valsalva [107]. The stress of pushing can also be minimized via forceps lift-out or vacuum extraction [108]. Although promising, further studies and multiple centers with a large cohort are needed to confirm these findings. After discussion of risks and benefits of each approach, physicians and patients can pursue an individualized decision, but when in doubt, current guidelines recommend C-section [5].

C-section Delivery: C-section delivery offers the ability to theoretically control the environment and avoid the liability of labor. However, clinicians should monitor for anesthesia-related and surgical complications, such as perioperative fluid shifts, surgical site infections, and post-operative complications such as ileus, atelectasis, or aspiration [21].

Anesthesia: Anesthesia with regional or neuro-axial blockade is preferred over general [104], as worse outcomes have been reported with general anesthesia compared to regional [10]. Epidural anesthesia is recommended to avoid pain-associated catecholamine surges [21]. However, a regional approach with spinal-epidural anesthesia unfortunately does not improve overall PAH-associated mortality during pregnancy [17].

Worse outcomes with general anesthesia may be secondary to intubation, which is not well tolerated in PAH. Intubation carries the risk of vasoplegia with induction and sedation, which can dramatically reduce preload and cardiac output. Positive pressure ventilation further decreases preload to the RV and increases RV afterload. In sum, intubation can rapidly lead to acute right heart failure, subsequent left heart failure secondary to intraventricular coupling, and circulatory collapse in patients with PAH [109]. Despite its potential drawbacks, general anesthesia may be beneficial for some patients such as those needing inhaled pulmonary vasodilators in a closed ventilatory system [106]. Inhaled nitric oxide can be beneficial by reducing RV afterload and improving RV cardiac output [110]. Notably, however, pulmonary vasodilators are less effective in severe, chronic pulmonary hypertension due to the longstanding fibrotic changes in pulmonary arteries [109].

### 3.6. Monitoring

Patients are generally monitored 24–48 h prior to planned inductions [70]. Non-invasive monitoring, such as blood pressure, heart rate, and oxygen saturation, is considered essential [111]. Serum studies, such as NT-proBNP, can be trended to inform clinical status [112,113]. Consensus pulmonary guidelines also recommend invasive monitoring with arterial and central venous catheters [5], but clinical judgement can be beneficial to tailor monitoring to severity of disease and escalate to invasive measures as needed. The use of PA catheters is controversial, especially given lack of clear benefit in outcomes [10], and so Swan–Ganz monitoring is currently not recommended for routine monitoring [5]. It may be worthwhile to pursue a less invasive approach initially, with the contingency for advanced therapies and interventions if hemodynamic challenges or complications arise. Point of care transthoracic echocardiogram is also beneficial for monitoring volume status and cardiac function [104].

### 3.7. Breastfeeding and Postpartum Management

Breastfeeding: Some institutions recommend against breastfeeding due to the risk of PAH medications crossing in the breastmilk [68]. However, some medications, such as sildenafil and calcium channel blockers, are considered at low risk for causing clinical harm to newborns despite crossing in breastmilk [114,115]. Breastfeeding can be decided through an individualized approach after discussion of risks and benefits.

### 3.8. Postpartum

All patients, even those who deliver without overt complications, are monitored closely in the immediate postpartum period. Especially for those with more severe disease, patients can be considered for treatment with IV prostaglandin for 48–72 h postpartum [21,116,117,118]. Monitoring is recommended for at least 48–72 h [95] but up to 5–14 days after birth [21,70,111,116]. The immediate postpartum period requires careful management of volume status, often requiring diuresis for a net negative balance [21]. At our institution, monitoring is completed through physical exam, proBNP, and echocardiogram as needed based on clinical symptoms. After discharge, patients are generally evaluated in the outpatient setting at 1-week, 3-weeks, 1-month, and 3-months [70]. A surveillance echocardiogram should be considered at 1-month postpartum [21].

**Figure 4:**
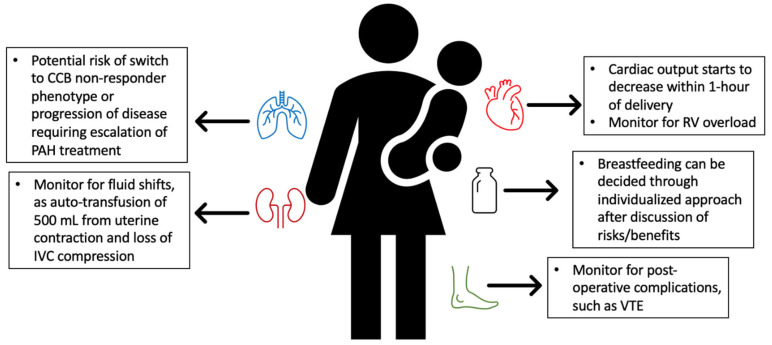
Postpartum changes in the patient with PAH. CCB = calcium channel blocker. PAH = pulmonary arterial hypertension. IVC = inferior vena cava. RV = right ventricle. VTE = venous thromboembolism.

There are many physiological changes in the immediate postpartum period which occur in all women immediately following delivery, but which have the potential to be detrimental to patients with PAH (Figure 4). The postpartum period is characterized by hemodynamic and fluid shifts, which can be harmful to a failing RV. Placental autotransfusion up to 300 to 500 mL [35,119] can overwhelm a tenuous RV. Alternatively, decreased venous return secondary to hemorrhage or vasovagal venodilation can stress a preload-dependent RV [21].

The most common cause of death in PAH during pregnancy is right heart failure [120]. If PAH progresses to RV failure, general principles include oxygen supplementation to avoid hypoxemia, fluid optimization through diuresis, and reduction of RV afterload [21]. Inotropes, such as dobutamine, can be considered for support of myocardial contractility [70]. Vasodilators like nitric oxide can be used to reduce PVR or RV afterload [84]. Based on our institutional experience, patients with severe PAH do not tolerate common obstetric complications, like chorioamnionitis (sepsis), preeclampsia (increased afterload), and postpartum hemorrhage (acute hypovolemia), and as such, it is important that obstetric practitioners look for warning signs of these complications.

Advanced therapies such as mechanical circulatory support can be considered for rapidly failing patients [104]. For example, cardiopulmonary bypass support has been employed for patients with acutely rising PA pressure and subsequent right heart failure to support them through C-section. Cardiopulmonary bypass can improve RV hemodynamics by unloading the pressure on RV, reducing pulmonary circulation pressures, and allowing for tight control of circulatory volumes to the mother and fetus [121]. Similarly, there are rare successful cases of veno-arterial ECMO use in the setting of right heart failure and hemodynamic collapse during pregnancy and delivery [122,123,124]. ECMO use is often limited by clinical trajectory, as it cannot be used indefinitely and generally serves as a bridge either to recovery or transplant.

More research is needed on the effects of pregnancy on PAH prognosis long-term. There are suggestions that pulmonary function may worsen after pregnancy [117]. Some patients have demonstrated need for escalating PAH treatment with subsequent pregnancies [21]. There are also some data that pregnancy may change disease phenotype. There are examples of patients with CCB-responder phenotype who converted to non-responder phenotype postpartum [125]. Further case reports and data are needed to understand the relationship of pregnancy and PAH beyond the immediate peripartum period. Additionally, there is some risk of ongoing right ventricular dysfunction and failure after a pregnancy in patients with PAH. Worsening right ventricular failure indicates poor long-term prognosis, and hence these individuals need to be closely monitored in the post-partum time frame.

## 4. Conclusions

Reproductive planning and contraceptive counseling are a routine part of cardiovascular care for patients with PAH. Patients with PAH are currently recommended against pregnancy due to concerning maternal and fetal outcomes, particularly in patients with severe disease, but there are cautiously optimistic, small study reports of improving outcomes, particularly in patients with milder disease. If pregnancy is pursued despite risk, patients with PAH benefit from care at a specialized center with a collaborative cardio-obstetrics team, including cardiopulmonary experts in PAH, maternal fetal medicine, and obstetric/cardiac anesthesia. A detailed pregnancy care plan can be developed in advance through multidisciplinary consultation. Future research and case report dissemination is needed to facilitate patient counseling on this subject and improve maternal and fetal outcomes for patients with PAH who pursue pregnancy.

## Figures and Tables

**Table 1:** Maternal cardiovascular risk assessments for patients with pulmonary hypertension.

Modified WHO Classification	Class IV: Extremely High–Risk Maternal Mortality and Morbidity (cardiomyopathy with LVEF < 30%, pulmonary hypertension, native severe coarctation, severe mitral and aortic stenosis) = 40–100% risk of maternal cardiovascular complications
CARPREG II Risk Predictors (Weighted risk score based on lesion, imaging parameters, and patient factors)	Pulmonary Hypertension (2) = 10% maternal cardiac complications risk

**Table 2:** Strengths and weaknesses of delivery methods for patients with PAH undergoing childbirth.

Delivery Method	Strengths	Weaknesses
Vaginal Delivery	Non-surgical (reduce perioperative risk of intubation and minimize risk of post-operative complications) Option for modified valsalva pushing to avoid vasovagal response Risk of valsalva can be reduced by assisted second stage delivery (forceps lift-out or vacuum extraction)	Risk of poorly controlled pain (leading to vasovagal spiral with catecholamine release) Increased cardiac output with active labor, which can overwhelm RV Risk of hypotension or other medication effect if planned induction
Cesarean Delivery	Guideline recommended method in patients with PAH Controlled environment Avoid lengthy labor Option for regional anesthesia	Perioperative risks (e.g. fluid shifts) and risk of post-operative complications (e.g. risk of ileus) Risk of surgical site infections Risks associated with intubation if general anesthesia required

## Data Availability

Not applicable.
